# Differential musculoskeletal outcome reporting in patients receiving bempedoic acid or atorvastatin: a disproportionality analysis using the EudraVigilance database

**DOI:** 10.3389/fphar.2025.1736657

**Published:** 2026-01-22

**Authors:** Gianluca Gazzaniga, Antonio Romio, Chiara Galuppi, Elena Gentile, Filomena Valentino, Luca De Toni, Michela Foschiatti, Michele Gringeri, Stefano D’Onghia, Danilo Menichelli, Daniele Pastori, Marco Scatigna, Diego Maria Michele Fornasari, Stefano Grosdani, Arianna Pani

**Affiliations:** 1 Department of Medical Biotechnology and Translational Medicine, Postgraduate School of Clinical Pharmacology and Toxicology, University of Milano, Milano, Italy; 2 Department of General Surgery, Surgical Specialty and Anesthesiology, Sapienza University of Rome, Rome, Italy; 3 Unit of Andrology and Reproductive Medicine, Department of Medicine, University of Padova, Padova, Italy; 4 Department of Medical and Cardiovascular Sciences, Sapienza University of Rome, Rome, Italy; 5 IRCCS Neuromed, Località Camerelle, Pozzilli, Isernia, Italy; 6 IRCCS Cardiologic Centre Monzino, Milan, Italy; 7 Department of Medical Biotechnology and Translational Medicine, University of Milano, Milano, Italy; 8 Department of Oncology and Hematology-Oncology, University of Milano, Milano, Italy

**Keywords:** adverse drug reactions, atorvastatin, bempedoic acid, cardiovascular, drug safety, pharmacovigilance, statins

## Abstract

**Background:**

Elevated low-density lipoprotein cholesterol (LDL-C) is a major risk factor for atherosclerotic cardiovascular disease. Statins are the first choice LDL-C-lowering drugs, but often associated with musculoskeletal disorders (MSDs), limiting adherence. Bempedoic acid (BA) is a newer LDL-C-lowering prodrug acting upstream of statins with limited muscle tissue activation, offering an alternative to statin-intolerant patients. However, recent evidence suggests a higher-than-expected rate of muscle-related adverse drug reactions (ADRs). This study compares muscle-related ADRs for BA and atorvastatin (ATO) using the European spontaneous reporting system.

**Methods:**

ADRs reports were extracted from the earliest available date to 30 June 2024 and categorized by patient demographics and ADR type. Disproportionality analysis via Reporting Odds Ratios (RORs) was performed to assess differences in muscle-related ADRs between BA and ATO.

**Results:**

A total of 78,930 ADR reports, respectively 2,667 for BA-only, 76,137 for ATO-only and 126 for coadministration, were analysed. MSDs were the most frequently reported events, significantly higher in BA-only than ATO-only recipients (ROR 2.25, 95% CI 2.08–2.43). Musculoskeletal and muscle discomfort showed the highest odds of association with BA (6.97, 95% CI 4.46–10.91 and 6.37, 95% CI 4.58–8.85, respectively). Conversely, more severe conditions such as creatine phosphokinase increase (ROR 0.44, 95% CI 0.34–0.57) and rhabdomyolysis (ROR 0.05, 95% CI 0.02–0.10) were more frequently reported for ATO-only recipients. Overall, muscle-related ADRs reported for BA showed lower severity.

**Conclusion:**

Using a Registry-based approach, we found increased odds of muscle-related ADR reports in BA recipients compared to ATO, although characterized by more favourable clinical outcomes. It is suggested to pay increased attention to consider drug-related causes of muscle symptoms when BA is used, particularly when in combination with statins.

## Introduction

Atherosclerotic cardiovascular disease (ASCVD), is a leading cause of mortality in Western Countries, accounting for approximately 18 million deaths per year worldwide, with more than four million of which in Europe alone (Guo et al., 2023). Several risk factors independently contribute to the onset of ASCVD, resulting in the increased probability of experiencing a cardiovascular event during the patient’s lifetime. Dyslipidemia and familial hypercholesterolaemia (FH) represents major risk factors for ACSVD since available epidemiological studies highlight the existence of log-linear relationship between plasma levels of low-density lipoprotein cholesterol (LDL-C) and ASCVD (Borén and Williams, 2016; Pećin et al., 2017; Ference et al., 2012; Holmes et al., 2015). Accordingly, LDL-C lowering strategies are pivotal for reducing ASCVD-related mortality (Silverman et al., 2016).

Statins are the first-choice drugs used in LDL-C lowering therapy. Statins’ pharmacodynamics (PD) rely on the competitive inhibition of hydroxymethyl-glutaryl-coenzyme A (HMG-CoA) reductase, associating with an average 30%–50% of LDL-C reduction, according to the different molecules, daily dosage and interindividual variability (Boekholdt et al., 2014). This translates to a 5-years average 22% reduction in major vascular events for each statin-associated 1 mmol/L LDL-C lowering (Baigent et al., 2010). Musculoskeletal disorders (MSD) are the most prevalent adverse drug reactions (ADR) associated with the use of statins, ranging from myalgia, with a reported prevalence of 10%–15%, with or without the increase of creatine kinase levels, to the rarest rhabdomyolysis, with a prevalence of 1-3 patients/100.000 per year (Bruckert et al., 2005; Davidson et al., 2006; Gupta et al., 2017). Statin-induced myopathy has been associated with decreased cell levels of ubiquinone, a component of the mitochondrial respiratory chain which is produced by the HMG-CoA pathway, resulting in impaired energy metabolism and cell death (Pallotti et al., 2021; Safitri et al., 2021). However, the impairment of protein prenylation, inhibition of complexes I and III of the electron transport chain, oxidative stress, downregulation of mitochondrial biogenesis, impairment of the insulin receptor/Akt/mTORC pathway and cytochrome c-related apoptosis pathways have also been involved in MSD onset (Bouitbir et al., 2020). Therapy discontinuation related to MSD, also known as “statins intolerance”, is a current clinical issue associated with their use. MSD are estimated to affect up to 27% of statin users, and up to 62% in those who discontinue the therapy (Cohen et al., 2012; Reith et al., 2022).

Other non-statin LDL-C lowering agents have been developed, among which bempedoic acid (BA) is one of the most recent, being approved for clinical use in the U.S. and in Europe in 2020. The PD of BA relies on the competitive inhibition of adenosine triphosphate–citrate lyase (ACL), an enzyme involved in cholesterol synthesis upstream of HMG-CoA reductase. The use of BA in mono-therapy is associated with an average LDL-C reduction by 18%–20% (Duarte Lau and Giugliano, 2023; Yasmin et al., 2024). Importantly, BA is a prodrug since it is activated by the hepatic enzyme very long-chain acyl-CoA synthetase-1 (ACSVL1), whose expression by skeletal muscle is reported as negligible (Zabielski et al., 2024). On this basis, the active form of BA is not detectable in skeletal muscle specimens and thus claimed as non-myotoxic (Ruscica et al., 2019). In addition, recent studies suggest BA as a possible agonist of monophosphate-activated protein kinase (AMPK), a master regulator of metabolism with different effects on energy homeostasis and sterol synthesis such as the inhibition of acetyl-CoA carboxylase (ACC) and HMG-CoA reductase (Clarke and Hardie, 1990; Foretz and Viollet, 2011; Pinkosky et al., 2016). BA is administered via the oral route and its current clinical indications are: i) in combination with a statin, or with a statin and other lipid-lowering therapies, in patients unable to achieve the target LDL-C levels with the maximally tolerated doses of either drugs ii) as monotherapy or in combination with other lipid-lowering therapies in patients intolerant to statins or when the use statin is contraindicated (Nissen et al., 2023).

Recent studies suggest a more complex scenario of BP-related ADRs, for which the occurrence of myalgia and gout appear as more frequent than that reported in available clinical trials (Laufs et al., 2019; De Filippo et al., 2023; Nissen et al., 2023; Bays et al., 2024). However, the frequency of Muscle-related ADR for BA in current clinical practice is under-investigated.

In this study, we aimed to evaluate the occurrence of ADRs reported for BA, particularly focusing on muscle-related ADRs, in comparison with those reported for the mainstay of statin drugs: atorvastatin (ATO). To this aim we adopted a Registry-based approach, using the European spontaneous reporting system to evaluate reported ADRs through disproportionality analysis.

## Methods

### Data source

This study was performed in agreement with the READUS-PV guidelines for disproportionality analysis (Fusaroli et al., 2024). The READUS-PV Checklist is available as Supplementary Table S1. The study was based on public registry data and did not involve collection of identifiable private information. Accordingly, the study required no approval by the local ethics committee (Postigo et al., 2018).

A retrospective analysis on suspected ADRs retrieved from the European Pharmacovigilance database (EudraVigilance), a database managed by the European Medicines Agency (EMA) for spontaneous reporting, was performed with publicly accessible data provided for transparency. Data reported for BA were compared with those of atorvastatin (ATO) as a reference cholesterol-lowering therapy. To this regard, available data show that ATO is the most prescribed statin drug worldwide and is burdened by an ADR-related risk of withdrawal not different from placebo-treated patients in short-term trials, similarly to available data for BA (Adams et al., 2015). In addition, no clear differential pattern of myotoxicity has been identified among available statins, allowing to consider ATO as a reliable reference drug in cholesterol lowering therapy (McAfee et al., 2006).

Data extraction was performed using the “line listing” function on the EMA’s online portal (www.adrreports.eu). Data searches were conducted for Individual Case Safety Reports (ICSRs). Inclusion criteria were any report, without time restrictions, associated with the following active pharmaceutical ingredients and “Suspect Drug”: bempedoic acid, including its combinations, and atorvastatin, including its combinations.

Data were retrieved within the period spanning from the earliest available reporting date, respectively 09 December 2002 for ATO-only and 22 May 2019 for BA-only, to 30 June 2024. Extracted variables included primary source qualification, primary source country, patient’s age and sex and details of reported ADRs.

### Descriptive analysis

Each ICSRs was firstly characterized for patient age, sex, primary source qualification, primary source country for regulatory purposes, number of reported preferred term (PT) and systemic organic classification (SOC). Data were analysed both overall and upon stratification for either BA-only recipients, ATO-only recipients and recipients of both. In order to identify relevant areas of signal detection for ADRs, each reported PT was mapped to its corresponding SOC. Both PTs and SOCs were defined according to the Medical Dictionary for Regulatory Activities (MedDRA, version 27_0, Medical Dictionary for Regulatory Activities Terminology (MedDRA) | NCBO BioPortal), a standardized medical terminology system structured into five hierarchical levels, with SOC representing the broadest classification and PTs being the second-most specific level.

Since Musculoskeletal and Connective Tissue Disorders SOC includes PTs, such as arthralgia, which are not univocally related to muscle conditions, a refinement approach was adopted. In addition to considering the Musculoskeletal and Connective Tissue Disorders SOC, a predefined group of specific muscle-related PTs (MR-PTs) was selected, including: myalgia, blood creatine phosphokinase increased, rhabdomyolysis, muscle spasms, muscular weakness, myopathy, musculoskeletal stiffness, muscle disorder, muscle discomfort, musculoskeletal pain, musculoskeletal discomfort, myositis and necrotizing myositis.

### Disproportionality analysis

All analyses were performed using R software (Version 4.2.3; R Development Core Team (LaZerte, 2021)). The Reporting Odds Ratio (ROR) and corresponding 95% confidence intervals (CIs) were calculated to assess the disproportionality of ADRs associated with BA and ATO, considering both SOCs and the predefined MR-PTs. ATO served as reference group. ROR was calculated by the ratio of reported rate of BA-associated ADRs with the rate of the same AD reported for ATO. Forest plots were used to visually represent RORs and their corresponding 95% CIs for both SOCs and selected PTs. Chi-squared test was used to assess differences in frequencies. Lastly, a subgroup analysis stratified by sex was conducted to assess potential sex-related differences in the reporting of ICSRs. P values < 0.05 were considered as significant.

## Results

### ICSRs characteristics

**FIGURE 1:**
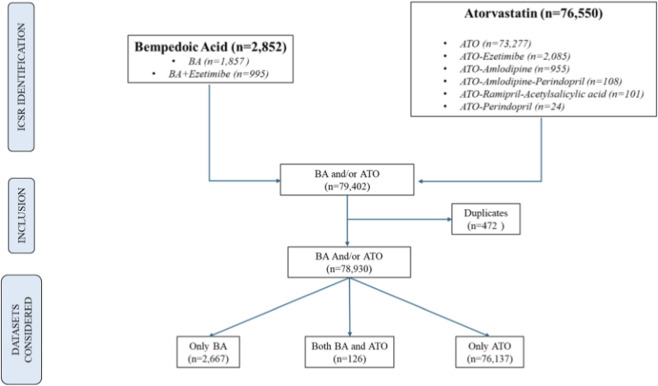
Flowchart of data extraction and dataset creation.

The flow chart of the data extraction process is summarized in Figure 1. Overall, 2,852 ICSRs were identified for BA and combined formulations, whilst 76,550 ICSRs were retrieved for ATO and combined formulations. Full details on retrieved combinations and time of extraction for each drug are available in Supplementary Table S2.

After removing duplicates, the final dataset comprised 78,930 reports whose details are reported in Table 1. In particular, 126 (0.16%) cases involved the co-administration of BA and ATO, 76,137 (96.5%) cases involved ATO or its combinations not including BA, and 2,667 (3.4%) cases involved BA or its combinations not including ATO. The majority of reports (64%) originated from healthcare professionals. Regarding geographical distribution, more than half (56%) of reports were from non-European Economic Area (EEA) Countries. However, focusing on BA cases, this trend was reversed since the vast majority of reports were from the EEA Countries (89%). Patient’s demographics showed that most cases involved adult patients in the age range 18–64 years (38%) and elderly patients in the age range 65–85 years (36%) patients, with a slight predominance of female sex across all groups.

**TABLE 1:** Individual Case Safety Reports (ICSRs) for Bempedoic Acid (BA) and Atorvastatin (ATO) reported in the EudraVigilance Spontaneous Reporting System from Initial Data Availability to 30 June 2024.

Variable	Overall^1^ (N = 78,930)	BA and ATO^1^ (N = 126)	ATO-only (N = 76,137)	BA-only (N = 2,667)
Primary source qualification				
Healthcare professional	50,872 (64%)	119 (94%)	48,584 (64%)	2,169 (81%)
Non-healthcare professional	27,612 (35%)	7 (5.6%)	27,107 (36%)	498 (19%)
Not specified	446 (0.6%)	0 (0%)	446 (0.6%)	0 (0%)
Primary source country for regulatory purposes				
EU area	34,658 (44%)	124 (98%)	32,159 (42%)	2,375 (89%)
Non-EU area	44,270 (56%)	2 (1.6%)	43,976 (58%)	292 (11%)
Not specified	2 (<0.1%)	0 (0%)	2 (<0.1%)	0 (0%)
Patient age group				
Pediatrics (<18 years)	171 (<0.1%)	0 (0%)	171 (<0.1%)	0 (0%)
Adults (18–64 years)	29,985 (38%)	23 (18%)	29,250 (38%)	712 (27%)
Elderly (65–85 years)	28,475 (36%)	22 (17%)	27,411 (36%)	1,042 (39%)
Very elderly (>85 years)	2,950 (3.7%)	2 (1.6%)	2,914 (3.8%)	34 (1.3%)
Not specified	17,349 (22%)	79 (63%)	16,391 (22%)	879 (33%)
Patient sex				
Female	37,893 (48%)	66 (52%)	36,388 (48%)	1,439 (54%)
Male	34,927 (44%)	57 (45%)	33,669 (44%)	1,201 (45%)
Not specified	6,110 (7.7%)	3 (2.4%)	6,080 (8.0%)	27 (1.0%)
Clinical disorder reported				
Blood and lymphatic system disorders	1,487 (1.9%)	0 (0%)	1,462 (1.9%)	25 (0.9%)
Cardiac disorders	2,916 (3.7%)	2 (1.6%)	2,852 (3.7%)	62 (2.3%)
Congenital, familial, and genetic disorders	50 (<0.1%)	0 (0%)	50 (<0.1%)	0 (0%)
Ear and labyrinth disorders	623 (0.8%)	0 (0%)	616 (0.8%)	7 (0.3%)
Endocrine disorders	141 (0.2%)	0 (0%)	141 (0.2%)	0 (0%)
Eye disorders	1,718 (2.2%)	2 (1.6%)	1,684 (2.2%)	32 (1.2%)
Gastrointestinal disorders	8,433 (11%)	30 (24%)	7,793 (10%)	610 (23%)
General disorders	12,845 (16%)	22 (17%)	12,267 (16%)	556 (21%)
Hepatobiliary disorders	7,220 (9.1%)	1 (0.8%)	7,190 (9.4%)	29 (1.1%)
Immune system disorders	5,786 (7.3%)	2 (1.6%)	5,701 (7.5%)	83 (3.1%)
Infections and infestations	1,170 (1.5%)	0 (0%)	1,155 (1.5%)	15 (0.6%)
Injury, poisoning, and procedural complications	8,075 (10%)	3 (2.4%)	7,881 (10%)	191 (7.2%)
Investigations	15,635 (20%)	16 (13%)	15,105 (20%)	514 (19%)
Metabolism and nutrition disorders	14,587 (18%)	3 (2.4%)	14,490 (19%)	94 (3.5%)
Musculoskeletal and connective tissue disorders	22,835 (29%)	110 (87%)	21,474 (28%)	1,251 (47%)
Nervous system disorders	14,705 (19%)	25 (20%)	14,180 (19%)	500 (19%)
Psychiatric disorders	5,250 (6.7%)	12 (9.5%)	5,101 (6.7%)	137 (5.1%)
Renal and urinary disorders	3,882 (4.9%)	1 (0.8%)	3,781 (5.0%)	100 (3.7%)
Respiratory, thoracic, and mediastinal disorders	6,169 (7.8%)	6 (4.8%)	6,029 (7.9%)	134 (5.0%)
Skin and subcutaneous tissue disorders	8,242 (10%)	7 (5.6%)	7,911 (10%)	324 (12%)
Vascular disorders	5,389 (6.8%)	1 (0.8%)	5,312 (7.0%)	76 (2.8%)

When ICSRs were stratified by SOC, musculoskeletal and connective tissue disorders SOC was the most frequently reported across all groups, accounting for 29% of all reports. Notably, this SOC was highly represented in both BA and ATO recipients (87% of reports) and in BA-only recipients (47% of reports) compared to ATO-only recipients (28%). Other commonly reported SOCs were investigations (20%), which includes various radiologic and laboratory tests, nervous system disorders (19%), metabolism and nutritional disorders (18%), gastrointestinal disorders (11%) and general disorders (16%).

**FIGURE 2:**
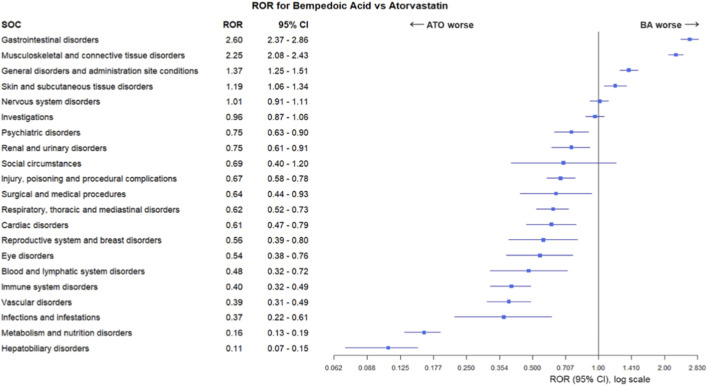
The Reporting Odds Ratio (ROR) analysis for systemic organic classification (SOC) of adverse drug reactions reported for bempedoic acid (BA) compared to atorvastatin (ATO).

Results of ROR analysis, comparing SOCs in BA-only cases and in ATO-only cases, are reported in Figure 2. Musculoskeletal and connective tissue disorders were significantly more reported in BA-only recipients compared to ATO-only recipients (ROR 2.25, 95% CI 2.08–2.43, p < 0.001). Similarly, BA-only recipients were more frequently reported for gastrointestinal disorders (ROR 2.60, 95% CI 2.37–2.86, p < 0.001), general disorders (ROR 1.37, 95% CI 1.25–1.51, p < 0.001) and skin and subcutaneous tissue disorders (ROR 1.19, 95% CI 1.06–1.34, p = 0.004). On the other hand, with the exception of nervous system disorders, investigations and social circumstances, most of the remaining SOCs had RORs and respective 95% CIs were suggestive of a disproportion of ADRs signals for ATO-only recipients, including metabolism and nutrition disorders (ROR 0.16, 95% CI 0.13–0.19, p < 0.001) and hepatobiliary disorders (ROR 0.11, 95% CI 0.07–0.15, p < 0.001).

### Muscle-related adverse events

The analysis of frequency, severity and reported outcome of predefined MR-PTs across the study groups are reported in Table 2. With a total of 11,332 reports (14%), myalgia was the overall most reported MR-PT, where BA-only recipients showed both the highest absolute prevalence, being reported in 24% of cases, and the highest prevalence of recovered/resolved cases compared to ATO-only recipients (64% versus 44%). Of note, cases of myalgia were reported in 68% of patients receiving both BA and ATO, with a prevalence of recovering/resolving of 77%. Cases of increased blood creatine phosphokinase levels, accounting for 5% of reports and showing no significant differential prevalence between ATO-only and BA-only recipients (5.0% and 2.3%, respectively), had a higher proportion of more severe cases, namely unresolved, resolved with sequelae or fatal, in ATO-only recipients (13.2% vs. 8.2% BA-only). Rhabdomyolysis showed the right-after reporting occurrence of 4.9%, where the highest reported fatality rate occurred in ATO-only (4.4%) compared to BA-only recipients (0.3%). Muscle spasms, muscular weakness, and myopathy were reported with lower frequencies of 3.1%, 2.6%, and 1.6%, respectively. The reported seriousness of these events was more pronounced in the ATO-only group with higher percentages of unresolved, resolved with sequelae and fatal cases. Other muscle-related PTs were relatively rare, each occurring in less than 1% of cases. However, muscle spasms were reported in 11% of patients receiving both BA and ATO, although with a high rate of recovering/resolution (79%).

**TABLE 2:** Severity of the adverse events-prespecified preferred term (PTs) in bempedoic acid (BA) or atorvastatin (ATO) recipients from the EudraVigilance retrieved cases.

PT	Overall, N = 78,930	Severity	BA and ATO, N = 126	ATO-only, N = 76.137	BA-only, N = 2.667
Myalgia	**11,332 (14%)**	​	**86 (68%)**	**10,615 (14%)**	**631 (24%)**
	​	Fatal	1 (1.2%)	16 (0.2%)	0 (0%)
	​	Recovered/Resolved with sequelae	0 (0%)	106 (1.0%)	1 (0.2%)
	​	Not recovered/Not resolved	4 (4.7%)	1,456 (14%)	57 (9.0%)
	​	Recovering/Resolving	2 (2.3%)	1,527 (14%)	69 (11%)
	​	Recovered/Resolved	66 (77%)	4,650 (44%)	405 (64%)
	​	Not specified/Unknown	13 (15%)	2,860 (27%)	99 (16%)
Increased blood creatine phosphokinase levels	**3,908 (5.0%)**	​	**10 (7.9%)**	**3,837 (5.0%)**	**61 (2.3%)**
	​	Fatal	0 (0%)	21 (0.5%)	0 (0%)
	​	Recovered/Resolved with sequelae	0 (0%)	27 (0.7%)	0 (0%)
	​	Not recovered/Not resolved	1 (10%)	449 (12%)	5 (8.2%)
	​	Recovering/Resolving	2 (20%)	817 (21%)	5 (8.2%)
	​	Recovered/Resolved	5 (50%)	1,218 (32%)	21 (34%)
	​	Not specified/Unknown	2 (20%)	1,305 (34%)	30 (49%)
Rhabdomyolysis	**3,833 (4.9%)**	​	**4 (3.2%)**	**3,822 (5.0%)**	**7 (0.3%)**
	​	Fatal	0 (0%)	169 (4.4%)	0 (0%)
	​	Recovered/Resolved with sequelae	0 (0%)	55 (1.4%)	0 (0%)
	​	Not recovered/Not resolved	1 (25%)	329 (8.6%)	0 (0%)
	​	Recovering/Resolving	2 (50%)	969 (25%)	1 (14%)
	​	Recovered/Resolved	0 (0%)	1,247 (33%)	2 (29%)
	​	Not specified/Unknown	1 (25%)	1,053 (28%)	4 (57%)
Muscle spasms	**2,438 (3.1%)**	​	**14 (11%)**	**2,280 (3.0%)**	**144 (5.4%)**
	​	Fatal	0 (0%)	0 (0%)	0 (0%)
	​	Recovered/Resolved with sequelae	0 (0%)	18 (0.8%)	0 (0%)
	​	Not recovered/Not resolved	3 (21%)	423 (19%)	24 (17%)
	​	Recovering/Resolving	0 (0%)	305 (13%)	17 (12%)
	​	Recovered/Resolved	11 (79%)	848 (37%)	75 (52%)
	​	Not specified/Unknown	0 (0%)	686 (30%)	28 (19%)
Muscular weakness	**2,021 (2.6%)**	​	**3 (2.4%)**	**1,967 (2.6%)**	**51 (1.9%)**
	​	Fatal	0 (0%)	11 (0.6%)	0 (0%)
	​	Recovered/Resolved with sequelae	0 (0%)	41 (2.1%)	0 (0%)
	​	Not recovered/Not resolved	1 (33%)	504 (26%)	14 (27%)
	​	Recovering/Resolving	1 (33%)	346 (18%)	10 (20%)
	​	Recovered/Resolved	1 (33%)	433 (22%)	20 (39%)
	​	Not specified/Unknown	0 (0%)	632 (32%)	7 (14%)
Myopathy	**1,265 (1.6%)**	​	**4 (3.2%)**	**1,239 (1.6%)**	**22 (0.8%)**
	​	Fatal	0 (0%)	13 (1.0%)	0 (0%)
	​	Recovered/Resolved with sequelae	0 (0%)	19 (1.5%)	0 (0%)
	​	Not recovered/Not resolved	1 (25%)	166 (13%)	0 (0%)
	​	Recovering/Resolving	0 (0%)	240 (19%)	0 (0%)
	​	Recovered/Resolved	3 (75%)	415 (33%)	11 (50%)
	​	Not specified/Unknown	0 (0%)	386 (31%)	11 (50%)
Musculoskeletal stiffness	**363 (0.5%)**	​	**0 (0%)**	**347 (0.5%)**	**16 (0.6%)**
	​	Fatal	0 (0%)	1 (0.3%)	0 (0%)
	​	Recovered/Resolved with sequelae	0 (0%)	7 (2.0%)	0 (0%)
	​	Not recovered/Not resolved	0 (0%)	95 (27%)	5 (31%)
	​	Recovering/Resolving	0 (0%)	36 (10%)	2 (13%)
	​	Recovered/Resolved	0 (0%)	86 (25%)	5 (31%)
	​	Not specified/Unknown	0 (0%)	122 (35%)	4 (25%)
Muscle disorder	**265 (0.3%)**	​	**0 (0%)**	**255 (0.3%)**	**10 (0.4%)**
	​	Fatal	0 (0%)	5 (2.0%)	0 (0%)
	​	Recovered/Resolved with sequelae	0 (0%)	1 (0.4%)	0 (0%)
	​	Not recovered/Not resolved	0 (0%)	47 (18%)	2 (20%)
	​	Recovering/Resolving	0 (0%)	28 (11%)	0 (0%)
	​	Recovered/Resolved	0 (0%)	45 (18%)	3 (30%)
	​	Not specified/Unknown	0 (0%)	129 (51%)	5 (50%)
Muscle discomfort	**255 (0.3%)**	​	**11 (8.7%)**	**200 (0.3%)**	**44 (1.6%)**
	​	Fatal	0 (0%)	0 (0%)	0 (0%)
	​	Recovered/Resolved with sequelae	0 (0%)	2 (1.0%)	0 (0%)
	​	Not recovered/Not resolved	1 (9.1%)	32 (16%)	7 (16%)
	​	Recovering/Resolving	2 (18%)	19 (9.5%)	3 (6.8%)
	​	Recovered/Resolved	7 (64%)	90 (45%)	26 (59%)
	​	Not specified/Unknown	1 (9.1%)	57 (29%)	8 (18%)
Musculoskeletal pain	**152 (0.2%)**	​	**0 (0%)**	**144 (0.2%)**	**8 (0.3%)**
	​	Fatal	0 (0%)	0 (0%)	0 (0%)
	​	Recovered/Resolved with sequelae	0 (0%)	1 (0.7%)	0 (0%)
	​	Not recovered/Not resolved	0 (0%)	30 (21%)	4 (50%)
	​	Recovering/Resolving	0 (0%)	22 (15%)	1 (13%)
	​	Recovered/Resolved	0 (0%)	34 (24%)	2 (25%)
	​	Not specified/Unknown	0 (0%)	57 (40%)	1 (13%)
Musculoskeletal discomfort	**126 (0.2%)**	​	**3 (2.4%)**	**99 (0.1%)**	**24 (0.9%)**
	​	Fatal	0 (0%)	0 (0%)	0 (0%)
	​	Recovered/Resolved with sequelae	0 (0%)	0 (0%)	0 (0%)
	​	Not recovered/Not resolved	0 (0%)	23 (23%)	3 (13%)
	​	Recovering/Resolving	2 (67%)	9 (9.1%)	2 (8.3%)
	​	Recovered/Resolved	1 (33%)	38 (38%)	13 (54%)
	​	Not specified/Unknown	0 (0%)	29 (29%)	6 (25%)
Myositis	**531 (0.7%)**	​	**0 (0%)**	**531 (0.7%)**	**0 (0%)**
	​	Fatal	0 (0%)	14 (2.6%)	0 (0%)
	​	Recovered/Resolved with sequelae	0 (0%)	6 (1.1%)	0 (0%)
	​	Not recovered/Not resolved	0 (0%)	93 (18%)	0 (0%)
	​	Recovering/Resolving	0 (0%)	118 (22%)	0 (0%)
	​	Recovered/Resolved	0 (0%)	101 (19%)	0 (0%)
	​	Not specified/Unknown	0 (0%)	199 (37%)	0 (0%)
Necrotising myositis	**163 (0.2%)**	​	**0 (0%)**	**163 (0.2%)**	**0 (0%)**
	​	Fatal	0 (0%)	2 (1.2%)	0 (0%)
	​	Recovered/Resolved with sequelae	0 (0%)	2 (1.2%)	0 (0%)
	​	Not recovered/Not resolved	0 (0%)	31 (19%)	0 (0%)
	​	Recovering/Resolving	0 (0%)	53 (33%)	0 (0%)
	​	Recovered/Resolved	0 (0%)	33 (20%)	0 (0%)
	​	Not specified/Unknown	0 (0%)	42 (26%)	0 (0%)

Values in bold represent the total for each PT.

**FIGURE 3:**
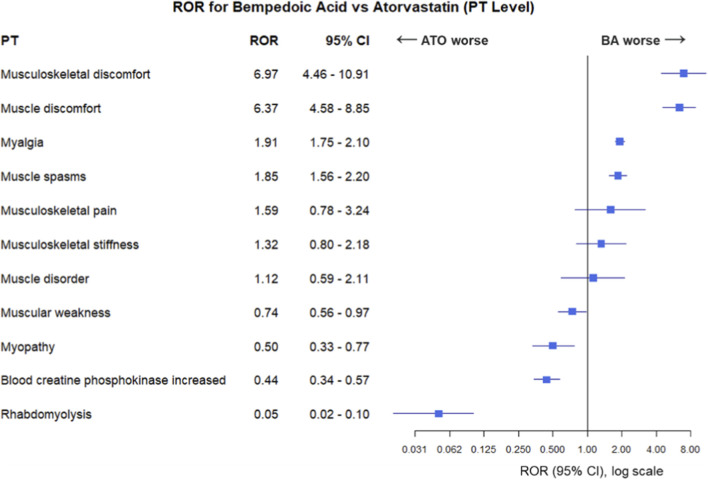
The Reporting Odds Ratio (ROR) analysis for muscle-related preferred terms (MR-PT) of adverse drug reactions reported for bempedoic acid (BA) compared to atorvastatin (ATO).

The ROR analysis for MR-PTs associated with BA-only compared to ATO-only recipients is reported in Figure 3. Reporting of musculoskeletal discomfort (ROR 6.97, 95% CI 4.46–10.91, p < 0.001) and muscle discomfort (ROR 6.37, 95% CI 4.58–8.85, p < 0.001) had the highest association with BA administration. Myalgia (ROR 1.91, 95% CI 1.75–2.10, p < 0.001) and muscle spasms (ROR 1.85, 95% CI 1.56–2.20, p < 0.001) also showed elevated reporting for BA-only recipients. In contrast, reporting of blood creatine phosphokinase increase (ROR 0.44, 95% CI 0.34–0.57, p < 0.001), and rhabdomyolysis (ROR 0.05, 95% CI 0.02–0.10, p < 0.001) were more frequently associated with ATO-only recipients.

### Subgroup analysis

After excluding ICSRs with unspecified sex, a total of 72,820 reports were stratified by sex. Descriptive characteristics of ICSRs for male and female patients are presented in Supplementary Table S3. The RORs for each SOC are provided in Supplementary Table S4. Overall, the trends were similar between sexes. In particular, for the Musculoskeletal and connective tissue disorders SOC, females exhibited a slightly higher ROR compared to males (ROR 2.28, 95% CI: 2.05–2.54 vs. ROR 1.98, 95% CI: 1.76–2.22, respectively).

Data on the seriousness of MR-PTs are shown in Supplementary Table S5, with no clinically meaningful differences emerging between sexes. Consistently, RORs and their 95% confidence intervals, depicted in Supplementary Figure S1, show similar trends for both males and females.

## Discussion

In this study, we compared ADR reports between Bempedoic Acid and Atorvastatin, a widely used statin class representative. We provide evidence that the use of BA associates with a more than doubled occurrence of reports depicting musculoskeletal or gastrointestinal disorder compared to ATO. In addition, BA showed an increased occurrence of reports related to general disorders, administration site conditions and nervous system disorders, compared to ATO. However, BA reports were characterized by lower severity and more favourable clinical outcomes, in terms of both lower rate of fatality and higher rate of resolution or recovery.

BA is a recent cholesterol lowering drug currently indicated, in monotherapy or in combination, in patients unable to achieve LDL-C target levels or in patients with intolerance/contraindication for the use statins because of its low myo-toxic potential (De Filippo et al., 2023; Duarte Lau and Giugliano, 2023). Indeed, in line with its mechanism of action, available meta-analyses of randomized clinical trials strongly support a low risk of musculoskeletal disorders associated with BA or even lower than that observed in placebo-treated patients (Goyal et al., 2024; Sayed et al., 2024). Furthermore, the reported therapy discontinuation for muscle-related ADRs was rare in BA recipients (Ballantyne et al., 2016; Thompson et al., 2016; Ray et al., 2019). However, recent evidence suggest that the use of BA is not devoid of the risk of myopathy. In fact, by the use of the Food and Drug Administration-ADR repository reporting system, Li et al. showed that musculoskeletal and connective tissue disorders are the most reported issues in BA recipients, with a ROR of 6.08 (CI 5.13–7.18), followed by nervous system disorders and hepatobiliary disorders (Li et al., 2024). Notably, the reported outcome severity, such as disability, hospitalization and life-threatening events, showed a prevalence of 10.47%, 8.90% and 1.57%, respectively, with a generally worse prognosis than that observed in our results, despite a fairly comparable demographic composition. Importantly, data from Li et al. were based on a lower number of reports compared to those available from our investigation, respectively 735 vs. 2667 BA-only recipients, which may imply a disproportionate representation of more serious ADRs in the two databases (Li et al., 2024). In addition, reporting sources from Li et al. were evenly distributed among Unites States, Germany and United Kingdom, whilst our dataset derived mostly from the EU area.

In order to provide a representative term of comparison for ADRs, we used ATO as a reference mainstay of cholesterol-lowering therapy. Indeed, ATO is the most prescribed statin drug worldwide, highly effective in patients with non-familiar hypercholesterolemia and whose risk of withdrawals associated with ADRs did not differ from placebo-treated patients in short-term trials (Adams et al., 2015). Overall, we found that muscle-related adverse events were more frequently reported in BA-only recipients but with a milder pattern of symptoms severity and clinical outcome. Differently, reported ADRs in ATO-only recipients showed a more severe pattern, being more frequently associated with rhabdomyolysis, increased CPK levels and a non-zero prevalence of fatal cases. This evidence opens up to various interpretative hypotheses. As previously reported for statins, long term HMGR inhibition associates with reduced levels of CoQ10, leading to mitochondrial dysfunction and muscle cell death (Bouitbir et al., 2020; Zeng et al., 2025). On the other hand, AMPK/ACC and AMPK/HMGR pathways modulation by BA is likely dependent on the proportion of free BA which is not converted in the active form bempedoyl-CoA (Clarke and Hardie, 1990; Foretz and Viollet, 2011; Pinkosky et al., 2016). Since a limited fraction of the drug remains unconverted, this likely associates with a less severe myotoxic effect than the one exerted by statins. In line with this hypothesis, we showed that the prevalence of MR-PTs reporting in patients receiving both BA and ATO was of 68%, underlining a possible cumulative effect on muscle outcomes based on the shared branches of AMPK-mediated pathways.

Alternatively, it might be hypothesized that BA-reporting suffered of the “alert-bias”, namely a higher propensity to report an adverse event associated with its use (Moore et al., 2003; Pariente et al., 2007). According to available clinical trials, a favourable myotoxicity profile has been clearly suggested for BA and, being anticipated as a safer alternative to statins, this may have led to an over-attention and reporting of ADRs for this new marketed drug. This phenomenon is not uncommon for newly marketed drugs, for which this heightened vigilance trend tends to decline over time (Moore et al., 2003). Moreover, randomized clinical trials, whilst representing the reference investigation addressing drug efficacy, only describe limited scenario of the possible ADRs a drug may generate in settings of clinical practice, given the limited sample size and stringent inclusion criteria. In this frame, deriving from pharmacovigilance data, findings here presented are suggested to have clinical relevance, deserving to consider drug-related causes of muscle symptoms, particularly even when BA is used in combination with statins. However, it is important to interpret these results with caution, deserving further investigation through more targeted tolerability studies.

We acknowledge some limitations to our findings. First, reporting bias, as previously mentioned, is a matter of concern. In addition, we recognize the limit of the selection of ATO as the sole comparator. However, adding additional comparators would have increased the already existing reporting disproportion between the two drug classes, leading to a possible underestimation of BA reporting. In addition, data sourced exclusively from the EudraVigilance database, providing limited access to clinical data to be used as confounding factors, restricting the generalizability of the findings. This is the case of dose stages of ATO and BA, which are considered as optional data and are rarely reported, and the patient’s age which generally reported as prevalence of a categorical variable <1 month, 12–17 years and >18 years. These represent additional limits to our findings. However, with respect to the more strictly geographical aspect, it should be noted that nearly 56% of reports were from no-EU area, providing some wide representativeness of our findings. In addition, we provided subgroup analysis by sex, showing no significant clustering of ADRs.

Finally, inherent limitations of disproportionality analysis must be taken into account, including the lack of critical information such as treatment duration, comorbidities and dosage, which prevents a comprehensive assessment of the long-term safety profile of the medications and the impossibility to establish causal relationships between drug exposure and ADRs. Further studies are warranted to assess how future pharmacovigilance signals trends will influence the reporting of ADRs related to BA and to investigate additional comparators.

In conclusion by the use of a Registry-based study, we showed an increased reporting of musculoskeletal-related ADR in BA recipients compared to ATO, although characterized by a lower severity outcome. Further studies are required to address the biological and clinical basis of this evidence.

## Data Availability

The raw data supporting the conclusions of this article will be made available by the authors, without undue reservation.

## Supplementary material

SUPPLEMENTARY FIGURE S1 Forest plots showing the Reporting Odds Ratios (RORs) and corresponding 95% Confidence Intervals (CIs) for prespecified muscle-related Preferred Terms (PT) associated with bempedoic acid compared to atorvastatin, stratified by sex: males **(A)** and females **(B)**.
