# Supplementary material for: Differential musculoskeletal outcome reporting in patients receiving bempedoic acid or atorvastatin: a disproportionality analysis using the EudraVigilance database
Source: Front Pharmacol. 2026 Jan 22;16:1736657. doi: 10.3389/fphar.2025.1736657 (PMC12872565; doi:10.3389/fphar.2025.1736657)
Supplement: Supplementary file 1 [file Table1.docx]

**Supplemental Table S1. The READUS-PV checklist**

| **Section and topic** | **Item**  **#** | **Checklist item** | **Location where item is reported**  **(Line)** |
| --- | --- | --- | --- |
| **Title** |  |  |  |
|  | 1a | If disproportionality analyses are a prominent component of the published study, the study should be identified as a “disproportionality analysis”. The type of data and name of the database(s) should be specified. | 2 |
|  | 1b | Report the name of adverse event(s) and/or drug(s) under study, when applicable. | 1 |
| **Introduction** |  |  |  |
| Background | 2a | Describe the drug(s) and its utilization, the nature of the adverse event(s) under study and its frequency, and the existing knowledge on the drug-event combination. | 82-95 |
|  | 2b | Specify the rationale for performing the analysis, e.g., as part of routine pharmacovigilance, to investigate an overall safety profile, or to assess a pre-specified hypothesis. | 116-123 |
|  | 2c | Explain why ICSR databases and disproportionality analysis are suitable to fill the knowledge gap. | 116-123 |
| Objectives | 3 | State specific objectives, identifying the adverse event(s), the drug(s), and the reference group, including any pre-specified hypothesis, if applicable. | 120-123 |
| **Methods** |  |  |  |
| Study design | 4a | Identify the study (i.e., “disproportionality analysis”) and the type of data used (e.g., “individual case safety reports”). | 128-130 |
|  | 4b | Provide an outline of the entire study design, including primary and sensitivity analyses performed, and other designs such as case-by-case analysis or literature review. | 132-137; 146-173 |
| Data description, access, and pre- processing | 5a | Specify the name of the database(s), the database(s) custodian, and the coverage. Specify the type/number of drugs included within the database and the thesaurus, taxonomies, or ontologies used for coding drugs and events. | 132-141 |
|  | 5b | Specify the extraction dates and describe and justify all choices used for data pre-processing, including any data transformation or exclusion, if appropriate. | 177-180 |
| Variables definition | 6a | Describe the study population, including any restriction. | 181-195 |
|  | 6b | Describe the nature and the meaning of key variables assessed in the work. | 155-160 |
|  | 6c | Specify and justify any grouping of drugs or events. For drugs, specify and justify whether active ingredients/trade names/salts were considered and/or the selected role. | 140-141; 155-160 |
|  | 6d | Describe any additional data source used, the type of data, and how they interact with ICSRs. | NA |
| Statistical methods | 7a | Present any descriptive analysis performed, specifying variables investigated, statistical tests, and significance thresholds. | 162-169 |
|  | 7b | Describe the measure(s) selected for the disproportionality analysis including any threshold used to identify signals of disproportionate reporting. Explain the reason for this choice if applicable. | 162-169 |
|  | 7c | Clearly describe any sensitivity analysis and any tool to control confounding, including any restriction, subgroup, stratification, adjustment, or interaction. | 171-173 |
|  | 7d | Specify the variables and methods used for the case-by-case analysis, including any algorithm or criteria used to assess causality, if performed. | NA |
|  | 7e | Specify any statistical methods used for other data sources. | NA |
| **Results** |  |  |  |
| Participants | 8a | Specify the number of individual case safety reports included at each stage, including reasons for exclusion. | 177-179 |
|  | 8b | Provide key demographic and clinical characteristics of cases, if possible comparing cases with any appropriate reference group. | 181-195 |
| Disproportionality analysis | 9 | Present all results including confidence intervals. Present also results of sensitivity analyses, if performed. | 196-204; 222-228; 230-239 |
| Case-by-case  analysis | 10 | Present the case-by-case analysis of key variables. Present the causality assessment, if applicable. | NA |
| **Discussion** |  |  |  |
| Key results | 11 | Discuss key results with reference to study objectives and contextualize them within the current literature and other consulted sources. Clearly discriminate between expected reactions and emerging safety signals. | 243-303 |
| External validity | 12a | Discuss the external validity of the results to the general population. | 295-297 |
|  | 12b | Discuss the potential relevance of results in clinical practice | 297-299 |
|  | 12c | Propose further study designs if applicable | 314-316 |
| Limitations | 13 | Present general limitations, making clear that disproportionality analysis alone cannot prove causation or measure incidence, and specific limitations, including confounding and reporting bias and efforts to mitigate them. | 307-317 |
| **Declarations** |  |  |  |
|  | 14a | Provide the source of funding/sponsorship and the role of the funders/sponsors for the present study and for any original study on which the present article is based. | 25-29 |
|  | 14b | Clearly identify potential commercial and intellectual conflicts of interest (e.g., link to any drug/event investigated, whether financial, legal action, or software used). | 31-32 |
|  | 14c | Declare any institutional approval needed or granted in the investigation. | 34-36 |
|  | 14d | Include a statement on data availability, code availability (including the version of the statistical software used), and protocol registration. | 34-36 |
