# Supplementary figures and images for: Differential musculoskeletal outcome reporting in patients receiving bempedoic acid or atorvastatin: a disproportionality analysis using the EudraVigilance database
Source: Front Pharmacol. 2026 Jan 22;16:1736657. doi: 10.3389/fphar.2025.1736657 (PMC12872565; doi:10.3389/fphar.2025.1736657)

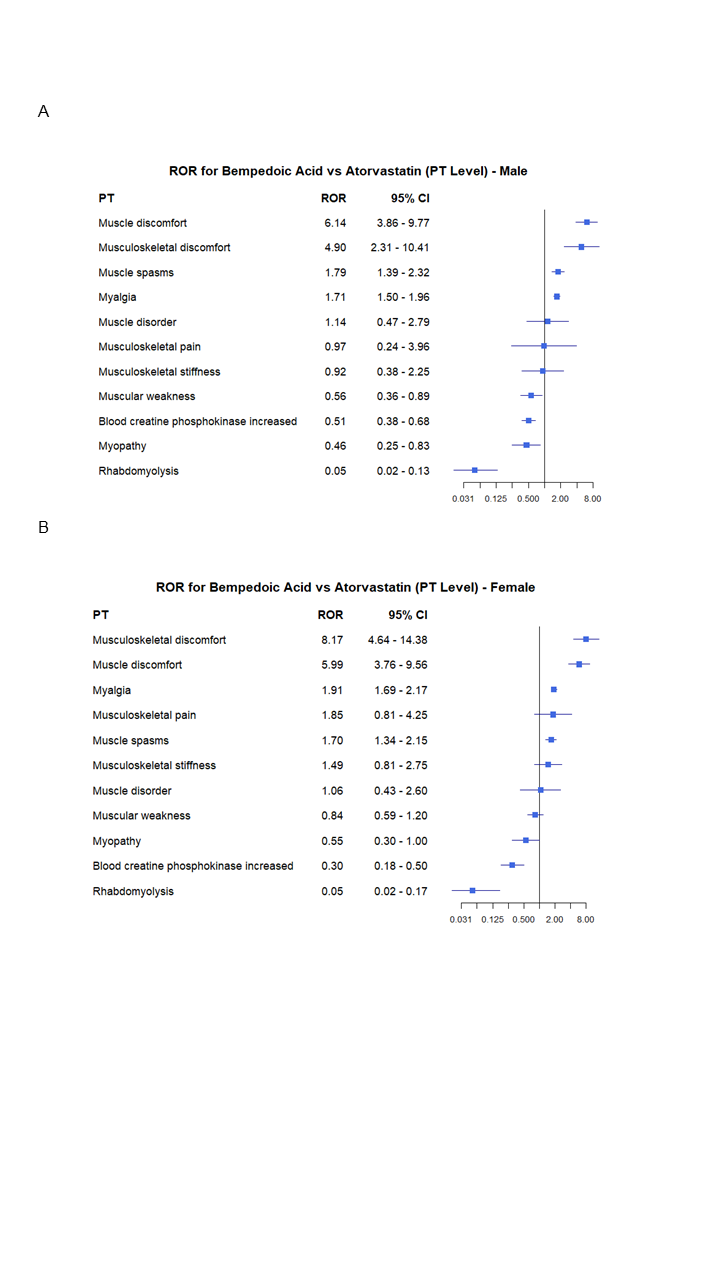

Supplement: Supplementary file 2 [file Image1.tif]
