# Supplementary material for: Differential musculoskeletal outcome reporting in patients receiving bempedoic acid or atorvastatin: a disproportionality analysis using the EudraVigilance database
Source: Front Pharmacol. 2026 Jan 22;16:1736657. doi: 10.3389/fphar.2025.1736657 (PMC12872565; doi:10.3389/fphar.2025.1736657)
Supplement: Supplementary file 3 [file Table2.docx]

**Supplemental Table S2. Retrieved combinations for bempedoic acid and atorvastatin**

| **Drug** | **N** | **From (date of first ICSR)** | **To (date of extraction)** |
| --- | --- | --- | --- |
| Bempedoic Acid | 1,857 | 22/05/2019 | 30/06/2024 |
| Bempedoic Acid-Ezetimibe | 995 | 04/12/2020 | 30/06/2024 |
| Atorvastatin | 73,277 | 09/12/2002 | 30/06/2024 |
| Atorvastatin-Ezetimibe | 2,085 | 18/03/2015 | 30/06/2024 |
| Atorvastatin-Amlodipine | 955 | 23/03/2005 | 30/06/2024 |
| Atorvastatin-Amlodipine-Perindopril | 108 | 14/09/2016 | 30/06/2024 |
| Atorvastatin-Ramipril-Acetylsalicylic acid | 101 | 17/08/2015 | 30/06/2024 |
| Atorvastatin-Perindopril | 24 | 05/11/2019 | 30/06/2024 |

ICSR, Individual Case Safety Report
