# Supplementary material for: Differential musculoskeletal outcome reporting in patients receiving bempedoic acid or atorvastatin: a disproportionality analysis using the EudraVigilance database
Source: Front Pharmacol. 2026 Jan 22;16:1736657. doi: 10.3389/fphar.2025.1736657 (PMC12872565; doi:10.3389/fphar.2025.1736657)
Supplement: Supplementary file 4 [file Table5.docx]

**Supplemental Table S5. Prespecified Muscle-Related Preferred Terms seriousness stratified by sex.**

|  | **Male (N = 34,972)** | | | | **Female (N = 37,893)** | | | |
| --- | --- | --- | --- | --- | --- | --- | --- | --- |
|  | **Overall**, N = 34,927 | **Both**, N = 57 | **Only ATO**, N = 33,669 | **Only BA**, N = 1,201 | **Overall**, N = 37,893 | **Both**, N = 66 | **Only ATO**, N = 36,388 | **Only BA**, N = 1,439 |
| **Myalgia** | 5,692 (16%) | 39 (68%) | 5,359 (16%) | 294 (24%) | 5,385 (14%) | 46 (70%) | 5,003 (14%) | 336 (23%) |
| Fatal | 8 (0.1%) | 0 (0%) | 8 (0.1%) | 0 (0%) | 7 (0.1%) | 1 (2.2%) | 6 (0.1%) | 0 (0%) |
| Recovered/Resolved With Sequelae | 49 (0.9%) | 0 (0%) | 48 (0.9%) | 1 (0.3%) | 56 (1.0%) | 0 (0%) | 56 (1.1%) | 0 (0%) |
| Not Recovered/Not Resolved | 784 (14%) | 1 (2.6%) | 760 (14%) | 23 (7.8%) | 716 (13%) | 3 (6.5%) | 679 (14%) | 34 (10%) |
| Recovering/Resolving | 875 (15%) | 1 (2.6%) | 844 (16%) | 30 (10%) | 698 (13%) | 1 (2.2%) | 658 (13%) | 39 (12%) |
| Recovered/Resolved | 2,494 (44%) | 31 (79%) | 2,281 (43%) | 182 (62%) | 2,536 (47%) | 35 (76%) | 2,279 (46%) | 222 (66%) |
| Not Specified/Unknown | 1,482 (26%) | 6 (15%) | 1,418 (26%) | 58 (20%) | 1,372 (25%) | 6 (13%) | 1,325 (26%) | 41 (12%) |
| **Blood creatine phosphokinase increased** | 2,452 (7.0%) | 6 (11%) | 2,401 (7.1%) | 45 (3.7%) | 1,254 (3.3%) | 2 (3.0%) | 1,237 (3.4%) | 15 (1.0%) |
| Fatal | 11 (0.4%) | 0 (0%) | 11 (0.5%) | 0 (0%) | 8 (0.6%) | 0 (0%) | 8 (0.6%) | 0 (0%) |
| Recovered/Resolved With Sequelae | 19 (0.8%) | 0 (0%) | 19 (0.8%) | 0 (0%) | 7 (0.6%) | 0 (0%) | 7 (0.6%) | 0 (0%) |
| Not Recovered/Not Resolved | 290 (12%) | 1 (17%) | 288 (12%) | 1 (2.2%) | 146 (12%) | 0 (0%) | 142 (11%) | 4 (27%) |
| Recovering/Resolving | 530 (22%) | 0 (0%) | 526 (22%) | 4 (8.9%) | 253 (20%) | 0 (0%) | 252 (20%) | 1 (6.7%) |
| Recovered/Resolved | 799 (33%) | 4 (67%) | 779 (32%) | 16 (36%) | 411 (33%) | 1 (50%) | 405 (33%) | 5 (33%) |
| Not Specified/Unknown | 803 (33%) | 1 (17%) | 778 (32%) | 24 (53%) | 429 (34%) | 1 (50%) | 423 (34%) | 5 (33%) |
| **Rhabdomyolysis** | 2,192 (6.3%) | 1 (1.8%) | 2,187 (6.5%) | 4 (0.3%) | 1,375 (3.6%) | 3 (4.5%) | 1,369 (3.8%) | 3 (0.2%) |
| Fatal | 104 (4.7%) | 0 (0%) | 104 (4.8%) | 0 (0%) | 52 (3.8%) | 0 (0%) | 52 (3.8%) | 0 (0%) |
| Recovered/Resolved With Sequelae | 33 (1.5%) | 0 (0%) | 33 (1.5%) | 0 (0%) | 21 (1.5%) | 0 (0%) | 21 (1.5%) | 0 (0%) |
| Not Recovered/Not Resolved | 193 (8.8%) | 0 (0%) | 193 (8.8%) | 0 (0%) | 126 (9.2%) | 1 (33%) | 125 (9.1%) | 0 (0%) |
| Recovering/Resolving | 573 (26%) | 0 (0%) | 572 (26%) | 1 (25%) | 372 (27%) | 2 (67%) | 370 (27%) | 0 (0%) |
| Recovered/Resolved | 754 (34%) | 0 (0%) | 753 (34%) | 1 (25%) | 449 (33%) | 0 (0%) | 448 (33%) | 1 (33%) |
| Not Specified/Unknown | 535 (24%) | 1 (100%) | 532 (24%) | 2 (50%) | 355 (26%) | 0 (0%) | 353 (26%) | 2 (67%) |
| **Muscle spasms** | 1,130 (3.2%) | 7 (12%) | 1,057 (3.1%) | 66 (5.5%) | 1,256 (3.3%) | 7 (11%) | 1,172 (3.2%) | 77 (5.4%) |
| Recovered/Resolved With Sequelae | 9 (0.8%) | 0 (0%) | 9 (0.9%) | 0 (0%) | 9 (0.7%) | 0 (0%) | 9 (0.8%) | 0 (0%) |
| Not Recovered/Not Resolved | 225 (20%) | 2 (29%) | 212 (20%) | 11 (17%) | 218 (17%) | 1 (14%) | 204 (17%) | 13 (17%) |
| Recovering/Resolving | 151 (13%) | 0 (0%) | 143 (14%) | 8 (12%) | 168 (13%) | 0 (0%) | 159 (14%) | 9 (12%) |
| Recovered/Resolved | 438 (39%) | 5 (71%) | 402 (38%) | 31 (47%) | 482 (38%) | 6 (86%) | 432 (37%) | 44 (57%) |
| Not Specified/Unknown | 307 (27%) | 0 (0%) | 291 (28%) | 16 (24%) | 379 (30%) | 0 (0%) | 368 (31%) | 11 (14%) |
| **Muscular weakness** | 956 (2.7%) | 2 (3.5%) | 935 (2.8%) | 19 (1.6%) | 993 (2.6%) | 1 (1.5%) | 960 (2.6%) | 32 (2.2%) |
| Fatal | 6 (0.6%) | 0 (0%) | 6 (0.6%) | 0 (0%) | 5 (0.5%) | 0 (0%) | 5 (0.5%) | 0 (0%) |
| Recovered/Resolved With Sequelae | 22 (2.3%) | 0 (0%) | 22 (2.4%) | 0 (0%) | 18 (1.8%) | 0 (0%) | 18 (1.9%) | 0 (0%) |
| Not Recovered/Not Resolved | 249 (26%) | 0 (0%) | 239 (26%) | 10 (53%) | 265 (27%) | 1 (100%) | 260 (27%) | 4 (13%) |
| Recovering/Resolving | 156 (16%) | 1 (50%) | 154 (16%) | 1 (5.3%) | 176 (18%) | 0 (0%) | 167 (17%) | 9 (28%) |
| Recovered/Resolved | 211 (22%) | 1 (50%) | 205 (22%) | 5 (26%) | 232 (23%) | 0 (0%) | 217 (23%) | 15 (47%) |
| Not Specified/Unknown | 312 (33%) | 0 (0%) | 309 (33%) | 3 (16%) | 297 (30%) | 0 (0%) | 293 (31%) | 4 (13%) |
| **Myopathy** | 681 (1.9%) | 3 (5.3%) | 667 (2.0%) | 11 (0.9%) | 515 (1.4%) | 1 (1.5%) | 503 (1.4%) | 11 (0.8%) |
| Fatal | 7 (1.0%) | 0 (0%) | 7 (1.0%) | 0 (0%) | 5 (1.0%) | 0 (0%) | 5 (1.0%) | 0 (0%) |
| Recovered/Resolved With Sequelae | 14 (2.1%) | 0 (0%) | 14 (2.1%) | 0 (0%) | 5 (1.0%) | 0 (0%) | 5 (1.0%) | 0 (0%) |
| Not Recovered/Not Resolved | 78 (11%) | 1 (33%) | 77 (12%) | 0 (0%) | 82 (16%) | 0 (0%) | 82 (16%) | 0 (0%) |
| Recovering/Resolving | 124 (18%) | 0 (0%) | 124 (19%) | 0 (0%) | 86 (17%) | 0 (0%) | 86 (17%) | 0 (0%) |
| Recovered/Resolved | 229 (34%) | 2 (67%) | 222 (33%) | 5 (45%) | 190 (37%) | 1 (100%) | 183 (36%) | 6 (55%) |
| Not Specified/Unknown | 229 (34%) | 0 (0%) | 223 (33%) | 6 (55%) | 147 (29%) | 0 (0%) | 142 (28%) | 5 (45%) |
| **Musculoskeletal stiffness** | 157 (0.4%) | 0 (0%) | 152 (0.5%) | 5 (0.4%) | 198 (0.5%) | 0 (0%) | 187 (0.5%) | 11 (0.8%) |
| Fatal | 0 (0%) | 0 (NA%) | 0 (0%) | 0 (0%) | 1 (0.5%) | 0 (NA%) | 1 (0.5%) | 0 (0%) |
| Recovered/Resolved With Sequelae | 3 (1.9%) | 0 (NA%) | 3 (2.0%) | 0 (0%) | 4 (2.0%) | 0 (NA%) | 4 (2.1%) | 0 (0%) |
| Not Recovered/Not Resolved | 43 (27%) | 0 (NA%) | 40 (26%) | 3 (60%) | 56 (28%) | 0 (NA%) | 54 (29%) | 2 (18%) |
| Recovering/Resolving | 19 (12%) | 0 (NA%) | 19 (13%) | 0 (0%) | 18 (9.1%) | 0 (NA%) | 16 (8.6%) | 2 (18%) |
| Recovered/Resolved | 43 (27%) | 0 (NA%) | 43 (28%) | 0 (0%) | 45 (23%) | 0 (NA%) | 40 (21%) | 5 (45%) |
| Not Specified/Unknown | 49 (31%) | 0 (NA%) | 47 (31%) | 2 (40%) | 74 (37%) | 0 (NA%) | 72 (39%) | 2 (18%) |
| **Muscle disorder** | 128 (0.4%) | 0 (0%) | 123 (0.4%) | 5 (0.4%) | 124 (0.3%) | 0 (0%) | 119 (0.3%) | 5 (0.3%) |
| Fatal | 4 (3.1%) | 0 (NA%) | 4 (3.3%) | 0 (0%) | 1 (0.8%) | 0 (NA%) | 1 (0.8%) | 0 (0%) |
| Recovered/Resolved With Sequelae | 1 (0.8%) | 0 (NA%) | 1 (0.8%) | 0 (0%) | 0 (0%) | 0 (NA%) | 0 (0%) | 0 (0%) |
| Not Recovered/Not Resolved | 26 (20%) | 0 (NA%) | 24 (20%) | 2 (40%) | 23 (19%) | 0 (NA%) | 23 (19%) | 0 (0%) |
| Recovering/Resolving | 16 (13%) | 0 (NA%) | 16 (13%) | 0 (0%) | 11 (8.9%) | 0 (NA%) | 11 (9.2%) | 0 (0%) |
| Recovered/Resolved | 24 (19%) | 0 (NA%) | 23 (19%) | 1 (20%) | 22 (18%) | 0 (NA%) | 20 (17%) | 2 (40%) |
| Not Specified/Unknown | 57 (45%) | 0 (NA%) | 55 (45%) | 2 (40% | 67 (54%) | 0 (NA%) | 64 (54%) | 3 (60%) |
| **Muscle discomfort** | 130 (0.4%) | 6 (11%) | 102 (0.3%) | 22 (1.8%) | 119 (0.3%) | 3 (4.5%) | 94 (0.3%) | 22 (1.5%) |
| Recovered/Resolved With Sequelae | 0 (0%) | 0 (0%) | 0 (0%) | 0 (0%) | 2 (1.7%) | 0 (0%) | 2 (2.1%) | 0 (0%) |
| Not Recovered/Not Resolved | 23 (18%) | 1 (17%) | 19 (19%) | 3 (14%) | 17 (14%) | 0 (0%) | 13 (14%) | 4 (18%) |
| Recovering/Resolving | 11 (8.5%) | 0 (0%) | 9 (8.8%) | 2 (9.1%) | 10 (8.4%) | 0 (0%) | 9 (9.6%) | 1 (4.5%) |
| Recovered/Resolved | 64 (49%) | 5 (83%) | 47 (46%) | 12 (55%) | 57 (48%) | 2 (67%) | 41 (44%) | 14 (64%) |
| Not Specified/Unknown | 32 (25%) | 0 (0%) | 27 (26%) | 5 (23%) | 33 (28%) | 1 (33%) | 29 (31%) | 3 (14%) |
| **Musculoskeletal pain** | 60 (0.2%) | 0 (0%) | 58 (0.2%) | 2 (0.2%) | 88 (0.2%) | 0 (0%) | 82 (0.2%) | 6 (0.4%) |
| Recovered/Resolved With Sequelae | 1 (1.7%) | 0 (NA%) | 1 (1.7%) | 0 (0%) | 0 (0%) | 0 (NA%) | 0 (0%) | 0 (0%) |
| Not Recovered/Not Resolved | 10 (17%) | 0 (NA%) | 9 (16%) | 1 (50%) | 23 (26%) | 0 (NA%) | 20 (24%) | 3 (50%) |
| Recovering/Resolving | 11 (18%) | 0 (NA%) | 11 (19%) | 0 (0%) | 12 (14%) | 0 (NA%) | 11 (13%) | 1 (17%) |
| Recovered/Resolved | 14 (23%) | 0 (NA%) | 14 (24%) | 0 (0%) | 22 (25%) | 0 (NA%) | 20 (24%) | 2 (33%) |
| Not Specified/Unknown | 24 (40%) | 0 (NA%) | 23 (40%) | 1 (50%) | 31 (35%) | 0 (NA%) | 31 (38%) | 0 (0%) |
| **Musculoskeletal discomfort** | 55 (0.2%) | 1 (1.8%) | 46 (0.1%) | 8 (0.7%) | 66 (0.2%) | 0 (0%) | 50 (0.1%) | 16 (1.1%) |
| Not Recovered/Not Resolved | 12 (22%) | 0 (0%) | 11 (24%) | 1 (13%) | 13 (20%) | 0 (NA%) | 11 (22%) | 2 (13%) |
| Recovering/Resolving | 5 (9.1%) | 0 (0%) | 5 (11%) | 0 (0%) | 6 (9.1%) | 0 (NA%) | 4 (8.0%) | 2 (13%) |
| Recovered/Resolved | 25 (45%) | 1 (100%) | 19 (41%) | 5 (63%) | 27 (41%) | 0 (NA%) | 19 (38%) | 8 (50%) |
| Not Specified/Unknown | 13 (24%) | 0 (0%) | 11 (24%) | 2 (25%) | 20 (30%) | 0 (NA%) | 16 (32%) | 4 (25%) |
| **Myositis** | 283 (0.8%) | 0 (0%) | 283 (0.8%) | 0 (0%) | 228 (0.6%) | 0 (0%) | 228 (0.6%) | 0 (0%) |
| Fatal | 8 (2.8%) | 0 (NA%) | 8 (2.8%) | 0 (NA%) | 6 (2.6%) | 0 (NA%) | 6 (2.6%) | 0 (NA%) |
| Recovered/Resolved With Sequelae | 3 (1.1%) | 0 (NA%) | 3 (1.1%) | 0 (NA%) | 3 (1.3%) | 0 (NA%) | 3 (1.3%) | 0 (NA%) |
| Not Recovered/Not Resolved | 52 (18%) | 0 (NA%) | 52 (18%) | 0 (NA%) | 39 (17%) | 0 (NA%) | 39 (17%) | 0 (NA%) |
| Recovering/Resolving | 64 (23%) | 0 (NA%) | 64 (23%) | 0 (NA%) | 51 (22%) | 0 (NA%) | 51 (22%) | 0 (NA%) |
| Recovered/Resolved | 53 (19%) | 0 (NA%) | 53 (19%) | 0 (NA%) | 45 (20%) | 0 (NA%) | 45 (20%) | 0 (NA%) |
| Not Specified/Unknown | 103 (36%) | 0 (NA%) | 103 (36%) | 0 (NA%) | 84 (37%) | 0 (NA%) | 84 (37%) | 0 (NA%) |
| **Necrotising myositis** | 92 (0.3%) | 0 (0%) | 92 (0.3%) | 0 (0%) | 67 (0.2%) | 0 (0%) | 67 (0.2%) | 0 (0%) |
| Fatal | 1 (1.1%) | 0 (NA%) | 1 (1.1%) | 0 (NA%) | 1 (1.5%) | 0 (NA%) | 1 (1.5%) | 0 (NA%) |
| Recovered/Resolved With Sequelae | 2 (2.2%) | 0 (NA%) | 2 (2.2%) | 0 (NA%) | 0 (0%) | 0 (NA%) | 0 (0%) | 0 (0%) |
| Not Recovered/Not Resolved | 22 (24%) | 0 (NA%) | 22 (24%) | 0 (NA%) | 9 (13%) | 0 (NA%) | 9 (13%) | 0 (NA%) |
| Recovering/Resolving | 33 (36%) | 0 (NA%) | 33 (36%) | 0 (NA%) | 20 (30%) | 0 (NA%) | 20 (30%) | 0 (NA%) |
| Recovered/Resolved | 19 (21%) | 0 (NA%) | 19 (21%) | 0 (NA%) | 13 (19%) | 0 (NA%) | 13 (19%) | 0 (NA%) |
| Not Specified/Unknown | 15 (16%) | 0 (NA%) | 15 (16%) | 0 (NA%) | 24 (36%) | 0 (NA%) | 24 (36%) | 0 (NA%) |
