# Supplementary material for: Differential musculoskeletal outcome reporting in patients receiving bempedoic acid or atorvastatin: a disproportionality analysis using the EudraVigilance database
Source: Front Pharmacol. 2026 Jan 22;16:1736657. doi: 10.3389/fphar.2025.1736657 (PMC12872565; doi:10.3389/fphar.2025.1736657)
Supplement: Supplementary file 5 [file Table3.docx]

**Supplemental Table S3. Individual Case Safety Reports for Bempedoic Acid and Atorvastatin stratified by sex**

| **Characteristics** | **Male (N = 34,972)** | | | | **Female (N = 37,893)** | | | |
| --- | --- | --- | --- | --- | --- | --- | --- | --- |
|  | **Overall**, N = 34,927*^1^* | **Both**, N = 57*^1^* | **Only ATO**, N = 33,669*^1^* | **Only BA**, N = 1,201*^1^* | **Overall**, N = 37,893*^1^* | **Both**, N = 66*^1^* | **Only ATO**, N = 36,388*^1^* | **Only BA**, N = 1,439*^1^* |
| **Primary Source Qualification** |  |  |  |  |  |  |  |  |
| Healthcare Professional | 26,068 (75%) | 55 (96%) | 25,002 (74%) | 1,011 (84%) | 22,637 (60%) | 61 (92%) | 21,439 (59%) | 1,137 (79%) |
| Non Healthcare Professional | 8,601 (25%) | 2 (3.5%) | 8,409 (25%) | 190 (16%) | 15,094 (40%) | 5 (7.6%) | 14,787 (41%) | 302 (21%) |
| Not Specified | 258 (0.7%) | 0 (0%) | 258 (0.8%) | 0 (0%) | 162 (0.4%) | 0 (0%) | 162 (0.4%) | 0 (0%) |
| **Primary Source Country for Regulatory Purposes** |  |  |  |  |  |  |  |  |
| European Economic Area | 18,023 (52%) | 57 (100%) | 16,890 (50%) | 1,076 (90%) | 16,008 (42%) | 64 (97%) | 14,670 (40%) | 1,274 (89%) |
| Non European Economic Area | 16,902 (48%) | 0 (0%) | 16,777 (50%) | 125 (10%) | 21,885 (58%) | 2 (3.0%) | 21,718 (60%) | 165 (11%) |
| Not Specified | 2 (<0.1%) | 0 (0%) | 2 (<0.1%) | 0 (0%) |  |  |  |  |
| **Patient Age Group** |  |  |  |  |  |  |  |  |
| 0-1 Month | 8 (<0.1%) | 0 (0%) | 8 (<0.1%) | 0 (0%) | 8 (<0.1%) | 0 (0%) | 8 (<0.1%) | 0 (0%) |
| 2 Months - 2 Years | 11 (<0.1%) | 0 (0%) | 11 (<0.1%) | 0 (0%) | 17 (<0.1%) | 0 (0%) | 17 (<0.1%) | 0 (0%) |
| 3-11 Years | 23 (<0.1%) | 0 (0%) | 23 (<0.1%) | 0 (0%) | 15 (<0.1%) | 0 (0%) | 15 (<0.1%) | 0 (0%) |
| 12-17 Years | 44 (0.1%) | 0 (0%) | 44 (0.1%) | 0 (0%) | 36 (<0.1%) | 0 (0%) | 36 (<0.1%) | 0 (0%) |
| 18-64 Years | 14,574 (42%) | 14 (25%) | 14,232 (42%) | 328 (27%) | 15,045 (40%) | 9 (14%) | 14,659 (40%) | 377 (26%) |
| 65-85 Years | 13,508 (39%) | 8 (14%) | 13,070 (39%) | 430 (36%) | 14,651 (39%) | 14 (21%) | 14,033 (39%) | 604 (42%) |
| More than 85 Years | 1,296 (3.7%) | 0 (0%) | 1,282 (3.8%) | 14 (1.2%) | 1,620 (4.3%) | 2 (3.0%) | 1,598 (4.4%) | 20 (1.4%) |
| Not Specified | 5,463 (16%) | 35 (61%) | 4,999 (15%) | 429 (36%) | 6,501 (17%) | 41 (62%) | 6,022 (17%) | 438 (30%) |
| **Blood and lymphatic system disorders** | 786 (2.3%) | 0 (0%) | 775 (2.3%) | 11 (0.9%) | 665 (1.8%) | 0 (0%) | 651 (1.8%) | 14 (1.0%) |
| **Cardiac disorders** | 1,442 (4.1%) | 2 (3.5%) | 1,422 (4.2%) | 18 (1.5%) | 1,389 (3.7%) | 0 (0%) | 1,346 (3.7%) | 43 (3.0%) |
| **Congenital, familial and genetic disorders** | 28 (<0.1%) | 0 (0%) | 28 (<0.1%) | 0 (0%) | 20 (<0.1%) | 0 (0%) | 20 (<0.1%) | 0 (0%) |
| **Ear and labyrinth disorders** | 266 (0.8%) | 0 (0%) | 264 (0.8%) | 2 (0.2%) | 338 (0.9%) | 0 (0%) | 333 (0.9%) | 5 (0.3%) |
| **Endocrine disorders** | 63 (0.2%) | 0 (0%) | 63 (0.2%) | 0 (0%) | 73 (0.2%) | 0 (0%) | 73 (0.2%) | 0 (0%) |
| **Eye disorders** | 550 (1.6%) | 1 (1.8%) | 536 (1.6%) | 13 (1.1%) | 1,120 (3.0%) | 1 (1.5%) | 1,100 (3.0%) | 19 (1.3%) |
| **Gastrointestinal disorders** | 3,505 (10%) | 12 (21%) | 3,267 (9.7%) | 226 (19%) | 4,703 (12%) | 17 (26%) | 4,313 (12%) | 373 (26%) |
| **General disorders and administration site conditions** | 5,947 (17%) | 9 (16%) | 5,702 (17%) | 236 (20%) | 6,430 (17%) | 13 (20%) | 6,102 (17%) | 315 (22%) |
| **Hepatobiliary disorders** | 3,797 (11%) | 0 (0%) | 3,788 (11%) | 9 (0.7%) | 3,301 (8.7%) | 1 (1.5%) | 3,281 (9.0%) | 19 (1.3%) |
| **Immune system disorders** | 2,569 (7.4%) | 1 (1.8%) | 2,540 (7.5%) | 28 (2.3%) | 2,979 (7.9%) | 1 (1.5%) | 2,925 (8.0%) | 53 (3.7%) |
| **Infections and infestations** | 507 (1.5%) | 0 (0%) | 502 (1.5%) | 5 (0.4%) | 635 (1.7%) | 0 (0%) | 625 (1.7%) | 10 (0.7%) |
| **Injury, poisoning and procedural complications** | 3,988 (11%) | 1 (1.8%) | 3,912 (12%) | 75 (6.2%) | 3,855 (10%) | 2 (3.0%) | 3,739 (10%) | 114 (7.9%) |
| **Investigations** | 8,244 (24%) | 9 (16%) | 7,970 (24%) | 265 (22%) | 6,731 (18%) | 5 (7.6%) | 6,482 (18%) | 244 (17%) |
| **Metabolism and nutrition disorders** | 2,106 (6.0%) | 0 (0%) | 2,068 (6.1%) | 38 (3.2%) | 8,882 (23%) | 3 (4.5%) | 8,823 (24%) | 56 (3.9%) |
| **Musculoskeletal and connective tissue disorders** | 11,353 (33%) | 50 (88%) | 10,726 (32%) | 577 (48%) | 10,703 (28%) | 57 (86%) | 9,980 (27%) | 666 (46%) |
| **Neoplasms benign, malignant and unspecified (incl cysts and polyps)** | 489 (1.4%) | 0 (0%) | 488 (1.4%) | 1 (<0.1%) | 555 (1.5%) | 0 (0%) | 549 (1.5%) | 6 (0.4%) |
| **Nervous system disorders** | 6,499 (19%) | 13 (23%) | 6,304 (19%) | 182 (15%) | 7,692 (20%) | 12 (18%) | 7,365 (20%) | 315 (22%) |
| **Pregnancy, puerperium and perinatal conditions** | 26 (<0.1%) | 0 (0%) | 26 (<0.1%) | 0 (0%) | 96 (0.3%) | 0 (0%) | 95 (0.3%) | 1 (<0.1%) |
| **Product issues** | 396 (1.1%) | 0 (0%) | 393 (1.2%) | 3 (0.2%) | 389 (1.0%) | 0 (0%) | 388 (1.1%) | 1 (<0.1%) |
| **Psychiatric disorders** | 2,263 (6.5%) | 8 (14%) | 2,201 (6.5%) | 54 (4.5%) | 2,864 (7.6%) | 4 (6.1%) | 2,777 (7.6%) | 83 (5.8%) |
| **Renal and urinary disorders** | 1,930 (5.5%) | 0 (0%) | 1,891 (5.6%) | 39 (3.2%) | 1,827 (4.8%) | 1 (1.5%) | 1,767 (4.9%) | 59 (4.1%) |
| **Reproductive system and breast disorders** | 992 (2.8%) | 0 (0%) | 979 (2.9%) | 13 (1.1%) | 535 (1.4%) | 1 (1.5%) | 517 (1.4%) | 17 (1.2%) |
| **Respiratory, thoracic and mediastinal disorders** | 2,916 (8.3%) | 4 (7.0%) | 2,856 (8.5%) | 56 (4.7%) | 3,103 (8.2%) | 2 (3.0%) | 3,024 (8.3%) | 77 (5.4%) |
| **Skin and subcutaneous tissue disorders** | 3,736 (11%) | 5 (8.8%) | 3,603 (11%) | 128 (11%) | 4,352 (11%) | 2 (3.0%) | 4,156 (11%) | 194 (13%) |
| **Social circumstances** | 235 (0.7%) | 1 (1.8%) | 229 (0.7%) | 5 (0.4%) | 298 (0.8%) | 0 (0%) | 291 (0.8%) | 7 (0.5%) |
| **Surgical and medical procedures** | 622 (1.8%) | 1 (1.8%) | 613 (1.8%) | 8 (0.7%) | 584 (1.5%) | 1 (1.5%) | 562 (1.5%) | 21 (1.5%) |
| **Vascular disorders** | 2,752 (7.9%) | 0 (0%) | 2,721 (8.1%) | 31 (2.6%) | 2,421 (6.4%) | 1 (1.5%) | 2,375 (6.5%) | 45 (3.1%) |

*^1^* n (%); Median (IQR). ATO, Atorvastatin; BA, Bempedoic Acid. ICSRs not reporting sex were excluded.
