# Supplementary material for: Differential musculoskeletal outcome reporting in patients receiving bempedoic acid or atorvastatin: a disproportionality analysis using the EudraVigilance database
Source: Front Pharmacol. 2026 Jan 22;16:1736657. doi: 10.3389/fphar.2025.1736657 (PMC12872565; doi:10.3389/fphar.2025.1736657)
Supplement: Supplementary file 6 [file Table4.docx]

**Supplemental Table S4. Reporting Odds Ratios for each System Organ Class stratified for sex**

|  | **Male** | | **Female** | |
| --- | --- | --- | --- | --- |
| **SOC** | **ROR** | **95% CI** | **ROR** | **95% CI** |
| Gastrointestinal disorders | 2.16 | 1.86 – 2.50 | 2.6 | 2.30 – 2.94 |
| Musculoskeletal and connective tissue disorders | 1.98 | 1.76 – 2.22 | 2.28 | 2.05 – 2.54 |
| General disorders and administration site conditions | 1.20 | 1.04 – 1.39 | 1.39 | 1.22 – 1.58 |
| Skin and subcutaneous tissue disorders | 1.00 | 0.83 – 1.20 | 1.21 | 1.04 – 1.41 |
| Investigations | 0.91 | 0.79 – 1.05 | 0.94 | 0.82 – 1.08 |
| Nervous system disorders | 0.78 | 0.66 – 0.91 | 1.10 | 0.97 – 1.25 |
| Eye disorders | 0.68 | 0.39 – 1.18 | 0.43 | 0.27 – 0.68 |
| Psychiatric disorders | 0.67 | 0.51 – 0.89 | 0.74 | 0.59 – 0.93 |
| Social circumstances | 0.61 | 0.25 – 1.48 | 0.61 | 0.29 – 1.29 |
| Renal and urinary disorders | 0.56 | 0.41 – 0.78 | 0.84 | 0.64 – 1.09 |
| Respiratory, thoracic and mediastinal disorders | 0.53 | 0.40 – 0.69 | 0.62 | 0.49 – 0.79 |
| Injury, poisoning and procedural complications | 0.51 | 0.40 – 0.64 | 0.75 | 0.62 – 0.91 |
| Metabolism and nutrition disorders | 0.50 | 0.36 – 0.69 | 0.13 | 0.10 – 0.17 |
| Blood and lymphatic system disorders | 0.39 | 0.22 – 0.71 | 0.54 | 0.32 – 0.92 |
| Reproductive system and breast disorders | 0.37 | 0.21 – 0.63 | 0.83 | 0.51 – 1.35 |
| Surgical and medical procedures | 0.36 | 0.18 – 0.73 | 0.94 | 0.61 – 1.46 |
| Cardiac disorders | 0.35 | 0.22 – 0.55 | 0.80 | 0.59 – 1.09 |
| Vascular disorders | 0.30 | 0.21 – 0.43 | 0.46 | 0.34 – 0.62 |
| Immune system disorders | 0.29 | 0.20 – 0.43 | 0.44 | 0.33 – 0.58 |
| Infections and infestations | 0.28 | 0.11 – 0.67 | 0.40 | 0.21 – 0.75 |
| Hepatobiliary disorders | 0.06 | 0.03 – 0.11 | 0.14 | 0.09 – 0.21 |

CI, Confidence Interval; ROR, Reporting Odds Ratio; SOC, System Organ Class
